# Resting-state functional connectivity and alexithymia: Preliminary predictive evidence

**DOI:** 10.1016/j.jad.2026.122208

**Published:** 2026-07-10

**Authors:** Anika Holton, Tianye Zhai, Elise Shealy, Samuel Lucero, Laura Murray, Kevin Noemer, Yihong Yang, Betty Jo Salmeron, Amy Janes

**Affiliations:** a Neuroimaging Research Branch, Intramural Research Program, National Institute on Drug Abuse, National Institutes of Health, 251 Bayview Blvd, Baltimore, MD, 21224, USA

**Keywords:** Alexithymia, Resting-state functional connectivity, Predictive modeling, Emotion regulation, Affective processing, Neural correlates

## Abstract

Alexithymia, defined by reduced emotional awareness, is prevalent in psychiatric and substance use disorders; however, its relationship with brain circuitry remains insufficiently understood. Investigating alexithymia in healthy adults enables identification of core brain networks underlying disrupted emotional awareness, independent of comorbid pathology, and may inform the development of targeted interventions. In this study, 150 healthy individuals were assessed using the Toronto Alexithymia Scale (TAS-20) and underwent resting-state functional magnetic resonance imaging. Predictive modeling was applied to whole-brain functional connectivity data to identify network patterns predictive of individual differences in alexithymia. Distinct brain patterns emerged for the TAS-20 total score and the Difficulty Describing Feelings (DDF) subscale. For total alexithymia, the right thalamus and left dorsolateral prefrontal cortex (dlPFC) were implicated. Higher total alexithymia was associated with reduced connectivity between the thalamus and posterior insula and sensorimotor regions, as well as decreased connectivity between the dlPFC and the cerebellum, medial temporal lobe, and frontal cortex. For the DDF subscale, the left premotor cortex (PMC) was identified as a central node, with higher DDF scores linked to reduced connectivity between the left PMC and both the anterior insula and dorsal anterior cingulate cortex. Overall, decreased connectivity among sensory integration, cognitive control, and motor planning networks modestly predicted core features of alexithymia. These findings provide preliminary evidence for the neural networks underlying individual differences in emotional processing and provide a framework for future research on vulnerability to psychiatric disorders and the development of targeted, circuit-based interventions.

## 1. Introduction

Alexithymia is defined by difficulties in identifying and describing feelings, a restricted imagination, and a pronounced externally oriented thinking style (Sifneos, 1973; Taylor et al., 1999). Although it is not formally recognized as a clinical diagnosis, thresholds for clinically significant levels have been established. Rather than a categorical condition, alexithymia is conceptualized as a continuous trait present throughout the general population, including among healthy individuals. Alexithymia severity is relatively stable and is associated with increased symptom severity across diverse mental health conditions (Baker et al., 2004; Beauchaine et al., 2007; Corstorphine et al., 2007; Fairburn et al., 2003; Ghalehban and Besharat, 2011; Honkalampi et al., 1999; Landstra et al., 2013; Leible and Snell, 2004; Loas et al., 1997; Mennin et al., 2005; Preece et al., 2022; Quam et al., 2024; Rude and McCarthy, 2003; Salminen et al., 2006; Sloan et al., 2017; Smyth et al., 2007; Tesio et al., 2018; Tull et al., 2009). Due to the strong association between alexithymic traits and various forms of psychopathology, the transdiagnostic clinical relevance of addressing alexithymia is widely acknowledged. However, the effectiveness of current therapeutic interventions for alexithymia remains limited (Ogrodniczuk et al., 2005; Ogrodniczuk et al., 2011; Parker et al., 1998; Tacon, 2008; Taylor et al., 1999). Identifying the neural mechanisms underlying alexithymia may clarify the limitations of existing interventions and inform the development of more effective, targeted treatments. Despite efforts in the literature to address these issues, two primary concerns persist.

First, although studies in clinical populations have yielded valuable insights into the neural correlates of alexithymia, their interpretation is frequently confounded by co-occurring mental health or substance use disorders, making it difficult to isolate the neural patterns specific to alexithymia. Additionally, much of this research relies on task-based paradigms, which measure brain activity only in response to specific task demands. For instance, during tasks involving the identification or observation of emotionally valenced stimuli, individuals with alexithymia show reduced activity in the anterior cingulate cortex (ACC) (Lee et al., 2021; Moriguchi et al., 2007), insula, amygdala (Silani et al., 2008), left dorsolateral prefrontal cortex (dlPFC), dorsal pons, cerebellum (Moriguchi et al., 2007), and posterior cingulate cortex (Mantani et al., 2005) compared to healthy controls. Task-based studies in healthy adults implicate similar brain regions. For example, the Difficulty Identifying Feelings (DIF) subscale of the Toronto Alexithymia Scale (TAS-20; Bagby et al., 1994a) has been associated with decreased right amygdala activity when observing emotionally salient stimuli (Kugel et al., 2008; Pouga et al., 2010). Additionally, an a priori analysis of speech intonation processing regions found that the Difficulty Describing Feelings (DDF) TAS-20 subscale was associated with decreased activity in the left superior temporal gyrus (STG) and bilateral amygdala (Goerlich-Dobre et al., 2013). Collectively, these findings indicate that alexithymia is associated with reduced reactivity in regions involved in affective stimulus processing and sensory integration in both clinical and healthy populations, particularly during introspection and mentalizing. However, the focus on a priori-defined regions related to specific tasks limits the generalizability of these findings. Because these effects are context-dependent, such studies highlight an interaction between alexithymia and task performance, leaving the intrinsic neural features of alexithymia unresolved and emphasizing the need for nontask-based functional magnetic resonance imaging (fMRI) approaches to assess alexithymia variance.

Resting-state functional connectivity (rsFC) provides a powerful framework for examining intrinsic brain organization independent of task demands. Nevertheless, most rsFC studies of alexithymia have relied on hypothesis-driven analyses restricted to a priori regions of interest derived from task-based paradigms. While this approach has yielded valuable insights, it inherently limits discovery and may fail to capture the distributed nature of alexithymia-related neural alterations. Across both clinical and non-clinical populations, elevated alexithymia has been linked to widespread disruptions involving limbic, executive control, and self-referential networks, including altered amygdala–prefrontal and insula–prefrontal connectivity, as well as disruptions of default mode network connectivity (Ho et al., 2016; Kim et al., 2020; Li et al., 2024; Liemburg et al., 2012; Sutherland et al., 2013). The heterogeneity and breadth of these findings suggest that alexithymia cannot be adequately characterized by isolated regional abnormalities. Instead, they point to a need for unbiased connectivity analyses capable of identifying network-level organization that may be overlooked by regionally constrained approaches. The present study directly addresses this gap by adopting a comprehensive, data-driven rsFC framework to define connectivity patterns predictive of alexithymia.

A comprehensive, network-level approach is needed to capture how alexithymia varies independently of specific tasks or diagnoses. Connectome Predictive Modeling (CPM) provides a data-driven framework that leverages whole-brain connectivity to identify neural signatures predictive of behaviors or traits such as alexithymia (Shen et al., 2017). The cross-validated and generalizable nature of CPM enables the isolation of intrinsic neural network features associated with alexithymia, facilitating the distinction between core, trait-based mechanisms and those influenced by tasks or comorbidities. In the present study, alexithymia was assessed using the well-validated Toronto Alexithymia Scale (TAS-20; Bagby et al., 1994a,b, Parker et al., 2003). In addition to the total score of the TAS-20, we also conducted an a priori follow up analysis focusing on the Difficulty Describing Feelings (DDF) subscale of the TAS-20. This subscale was selected due to its demonstrated internal consistency in psychometric evaluations (Preece et al., 2017), its predictive value for substance use (Sutherland et al., 2022), its interaction between network connectivity and psychiatric disorders, such as substance use disorder (Liang et al., 2015) and its temporal stability during alcohol withdrawal (de Timary et al., 2008). Collectively, these findings indicate that the DDF subscale represents a psychometrically robust and clinically relevant dimension, which may have meaningful neurobiological mechanisms distinct from the total score. Accordingly, we applied a CPM-informed prediction modeling approach in a healthy sample to clarify the neurobiological basis of alexithymia (Total and DDF), independent of comorbid conditions.

## 2. Materials and methods

### 2.1. Participants

150 healthy participants were included who took part in a broad characterization study at the National Institute on Drug Abuse Intramural Research Program (NIDA-IRP) in Baltimore, Maryland, which aimed to gather a uniform set of assessments for deep phenotyping of all participants in NIDA imaging studies. The present analyses were conducted cross-sectionally using a single assessment time point from each participant. These participants were drawn from the control groups for ongoing studies of cocaine users, smokers, and those with schizophrenia. The study was approved by the institutional review board of the National Institutes of Health, in accordance with the Declaration of Helsinki. Written informed consent was obtained from each participant. Study exclusion criteria included major medical disorders that might affect fMRI measures (e.g., diabetes) assessed with medical history, physical exam, and laboratory assessments including urine toxicology, complete blood count, basic chemistry and liver functions, thyroid function, and an erythrocyte sedimentation rate. Study exclusion criteria also included any psychiatric disorders. Psychiatric disorders were excluded for following standardized assessment (computerized SCID with follow-up clinical interview, a MINI, or a standard SCID interview, depending on when in the course of data gathering the participant was assessed). The analytic sample was composed of participants without substance use disorders and all participants tested negative for drug use prior to participation. Data used in the current study were obtained between November 2011 and February 2022.

### 2.2. MRI acquisition

MRI scans were conducted using a Siemens 3 T scanner (Trio, Siemens, Erlangen, Germany). Anatomical images were acquired using a 3D magnetization-prepared rapid gradient-echo sequence (MPRAGE) T1-weighted sequence (TE = 3.51 ms, TR = 1.9 s, flip angle = 9°, spatial resolution = 1 mm × 1 mm × 1 mm). Six minutes of whole-brain blood-oxygen-level-dependent (BOLD) resting-state fMRI data were acquired in the axial plane parallel to the AC-PC line using a single-shot, echoplanar imaging sequence (TE = 27 ms, TR = 2 s, flip angle = 78°, spatial resolution = 3.44 mm × 3.44 mm × 4 mm with no gap). Participants were instructed to keep their heads still, eyes open, and not to think of anything in particular during the resting-state scan.

### 2.3. Analytical approach for alexithymia prediction modeling

Our current analysis utilized the prediction modeling pipeline described previously (Zhai et al., 2021), which is partially adapted and modified from the ‘connectome-based predictive modeling (CPM)’ by Shen et al. (2017). Generally, our analytical flow consisted of four conceptual steps: 1. Image preprocessing; 2. Region-of-Interest (ROI) generation and functional connectivity calculation; 3. Voxel-wise regression and composite indices generation; 4. Optimally fit composite indices from all applicable ROIs into the final prediction model. These procedural steps are illustrated in Fig. S1 and described in detail below.

#### 1. Image preprocessing

Raw MRI DICOM data was converted to the BIDS compatible structure/format with bidskit (v2022.5.24a, https://github.com/jmtyszka/bidskit). The MRI preprocessing was conducted using fMRIPrep (v22.1.0, https://fmriprep.org/en/22.1.0/) and AFNI (v20.2.06, http://afni.nimh.nih.gov/afni/) , which included discarding the first 3 volumes, slice timing correction, volume registration, head motion (HM) estimation, and spatial normalization to MNI space. Head motion was also evaluated at the volume-by-volume level to further control image quality by using pair-wise frame-wise displacement calculated with the Euclidean distance (*1d_tool.py*, AFNI). Volumes with displacement > 0.35 mm were censored (along with the volumes from the previous TR). Participants were excluded if their mean head motion across volumes was greater than 0.3 mm, or if their percentage of censored volumes exceeded 30%. Principal components of the signal from the white matter and cerebrospinal fluid were also estimated (*3dmaskSVD*, AFNI) for subsequent GLM based denoising (Behzadi et al., 2007). A set of band-pass filter regressors was also generated via discrete sine/cosine transform to select low frequency fluctuations within the range of 0.01–0.1 Hz (*1dBport*, AFNI). After all noise regressors were estimated/generated, a GLM that involved pre-whitening to properly model the temporal autocorrelation during nuisance regression (*3dREMLfit*, AFNI) was conducted (Bright et al., 2017; Olszowy et al., 2019), which incorporated all regressors in a single GLM step to avoid re-introducing artifacts into fMRI data (Lindquist et al., 2019; Zhai et al., 2026). Finally, the data was smoothed with 6 mm FWHM for subsequent analyses.

#### 2. Seed ROI generation and functional connectivity calculation

To explore the neural basis of alexithymia and identify candidate regions for further functional connectivity analyses, we first calculated whole-brain degree centrality (*3dDegreeCentrality*, AFNI). We then examined how these centrality measures related to alexithymia, as measured by the TAS-20 total score and DDF subscale (*3dttest*++, AFNI). Significance level was set at voxel-level *p* < 0.001, and cluster > 768 mm^3^ with cluster forming method of Nearest Neighbor 1 (i.e., face-touching-face for voxels). Regions showing significant associations with alexithymia were designated as seed ROIs for subsequent connectivity analyses, allowing us to investigate how connectivity patterns of these key regions relate to individual differences in alexithymia. The above seed ROI generation procedure was conducted on the full sample. For each seed ROI, a cross-correlation coefficient (CC) map was generated for each participant by correlating the time course of the seed ROI with that of each voxel of the whole brain (*3dDeconvolve*, AFNI). Fisher’s Z-transformation was then applied to the CC maps resulting in z maps that were used in subsequent regression analyses.

#### 3. Voxel-wise regression and composite indices generation

Voxel-wise regression was conducted to explore the relationship between the functional connectivity of these seed ROIs and alexithymia, with the factors of age, sex, and head movement during the fMRI scan as covariates:

$$rsFC=\beta_{\text{age}}Age+\beta_{\text{sex}}Sex+\beta_{hm}HM+\beta_{\text{alex}}Alex+\varepsilon .$$

The beta coefficient for each voxel was estimated to generate whole-brain beta maps. These maps were thresholded at *p* < 0.005 and used to identify: 1. a spatial pattern of ‘protective’ circuits, consisting of voxels with negative beta coefficient, which associate higher functional connectivity with lower alexithymia score, and a pattern of ‘risk’ circuits that are comprised of voxels with positive beta coefficient, which associate higher functional connectivity with higher alexithymia score; and 2. Two composite predictive indices, protective- and risk-index (indexP and indexR, respectively), with linear summation of the functional connectivity values of all voxels within the respective circuits. These indices were then used to construct the final prediction model.

#### 4. Cross-validation and the final prediction model

The procedures described in step 3 (C to G in Fig. S1) were implemented within a cross-validation framework. In each iteration, data from one participant were excluded from model estimation, and the resulting model was then used to predict the alexithymia score for the excluded participant. Within this framework, the predictive indices (indexP and indexR) of all ROIs were optimally fit into a final prediction model, with age, sex, and head movement during the fMRI scan included as covariates:

$$\text{Alexithymia}=\beta_{P1}\text{indexP}1+\beta_{R1}\text{indexR}1+\beta_{P2}\text{indexP}2+\beta_{R2}\text{indexR}2+\ldots +\beta_{\text{age}}\text{Age}+\beta_{\text{sex}}\text{Sex}+\beta_{hm}HM+\varepsilon$$

Because the identified circuits varied in size, a one-dimensional grid search was performed to determine the optimal circuit size threshold for combining indices of all ROIs into the final prediction model. This approach balanced model complexity and predictive accuracy by systematically evaluating how different circuit size thresholds affected performance. For each threshold, the mean squared error (MSE) between predicted and measured alexithymia scores was calculated, and the combination of composite indices with the lowest MSE was selected as the final prediction model. Model performance was assessed using linear regression between the measured and predicted TAS-20 scores within the Leave-One-Out (LOO) cross-validation framework. To visualize the brain topology of the final prediction model, beta maps from all LOO iterations were binarized and stacked together. A 75% overlap threshold was applied to generate a group-level heat map, highlighting the protective and risk circuits most consistently associated with alexithymia. The statistical significance of the final prediction model was determined nonparametrically using a permutation test with 10,000 repetitions, in which predictor and outcome pairs were randomly shuffled to generate the empirical null distribution of the MSE. The *p*-value was derived based on the ranking of the actual MSE within this empirical null distribution. Predictive performance was further evaluated using linear regression between the measured alexithymia scores and those predicted by the final model.

## 3. Results

### 3.1. Demographic and alexithymia evaluation

Of the 150 participants from our cohort, one was discarded due to excessive head motion during the resting-state fMRI scan, leaving 149 in the final analyses. As shown in Table 1, the cohort included 81 females and 68 males, with a mean (SD) age of 31.37 (10.03) years old (range, 18–58). Of these participants, 82 (55.0%) were African American, 48 (32.2%) White, 13 (8.7%) Asian, 5 (3.4%) Mixed, and 1 (0.7%) American Indian/Alaska Native. The Mean alexithymia scores (SD) were Total score: 36.58 (8.57), Difficulty Identifying Feelings (DIF) subscale score: 9.27 (3.47), Difficulty Describing Feelings (DDF) subscale score: 9.30 (3.92), and Externally Oriented Thinking (EOT) subscale score: 18.01 (4.18).

### 3.2. Seed ROIs for functional connectivity calculation

**Fig. 1.**
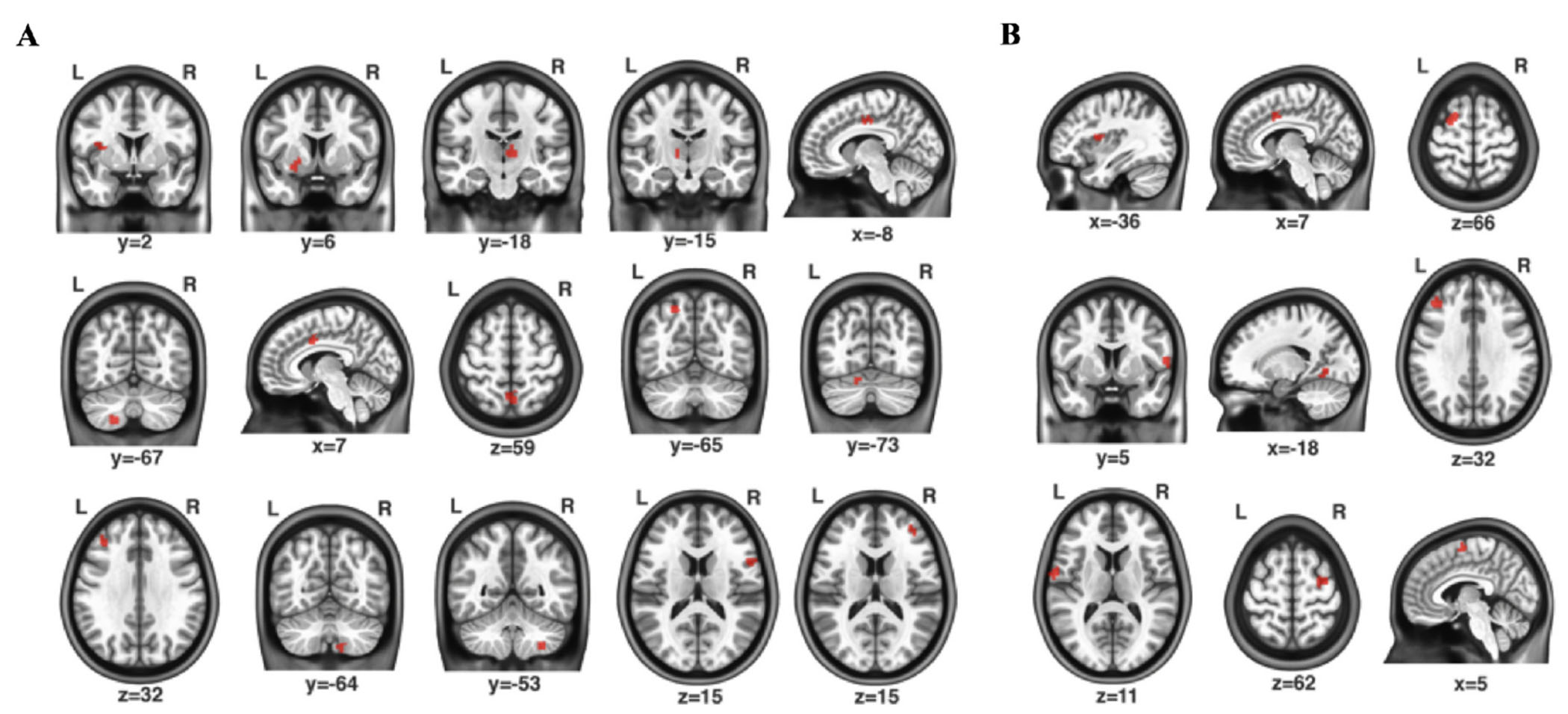
Seed ROIs identified from the correlation between whole brain degree-centrality and TAS-20 Total score (A) and DDF subscore (B).

After correction for multiple comparisons (*p* < 0.001, α < 0.05), spatial patterns with multiple significant clusters were identified in the correlations between degree centrality maps for both the Total score and the DDF subscale (Fig. S2). These patterns informed the definition of 15 seed ROIs for the Total score and 9 for the DDF subscale (Tables 2, 3, and Fig. 1).

### 3.3. Prediction modeling and the underlying functional circuits

Prediction models were constructed using the 15 and 9 seed ROIs for the TAS-20 Total and DDF scores, respectively. These models identified functional connectivity profiles predictive of alexithymia.

**Fig. 2.**
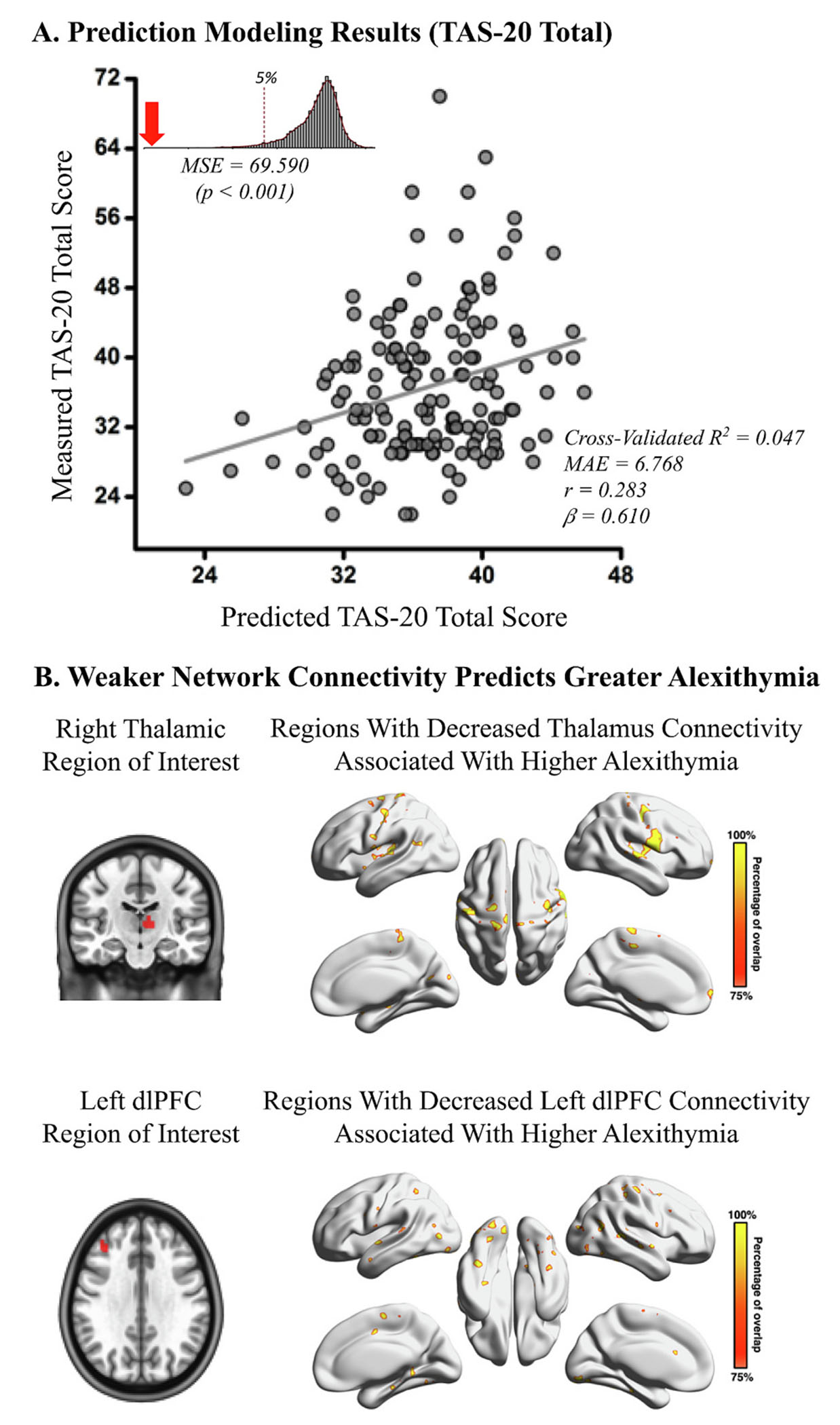
Prediction modeling and functional circuits predicting the TAS-20 Total score. Panel A shows the statistical significance of the final prediction model via permutation, and the prediction performance assessed with a linear regression between the predicted vs. the measured TAS-20 Total score. Panel B shows the surviving ROIs as well as the functional circuits that underlie the prediction model. Abbreviations: TAS, Toronto Alexithymia Scale; ROI, region-of-interest.

For the Total score, the optimal prediction model minimized the MSE between the predicted and measured values. The optimal model was achieved when the circuit size threshold ranged from 4200 to 5400 voxels. The model significantly predicted the Total score (*p* < 0.001), with linear regression between predicted and observed Total scores yielding *r* = 0.283, β = 0.610, cross-validated R^2^ of 0.047 and a mean absolute error (MAE) of 6.768 (Fig. 2A). The final model identified two networks: one originating from the right thalamus and another from the dlPFC (BA 46). The right thalamus network indicated that increased total alexithymia was associated with reduced connectivity between the right thalamus and regions of the sensorimotor system, as well as the posterior insula (pIns). The dlPFC network showed that greater total alexithymia was associated with reduced dlPFC connectivity with the left cerebellum (VII), bilateral cerebellum (VI), right frontal eye field (FEF; BA8), right precentral/postcentral gyrus, and temporal regions including the fusiform face area (FFA; BA37), hippocampus/parahippocampal areas, middle temporal gyrus (MTG), superior temporal gyrus (STG), and the right temporal-parietal junction (TPJ) (Fig. 2B).

**Fig. 3.**
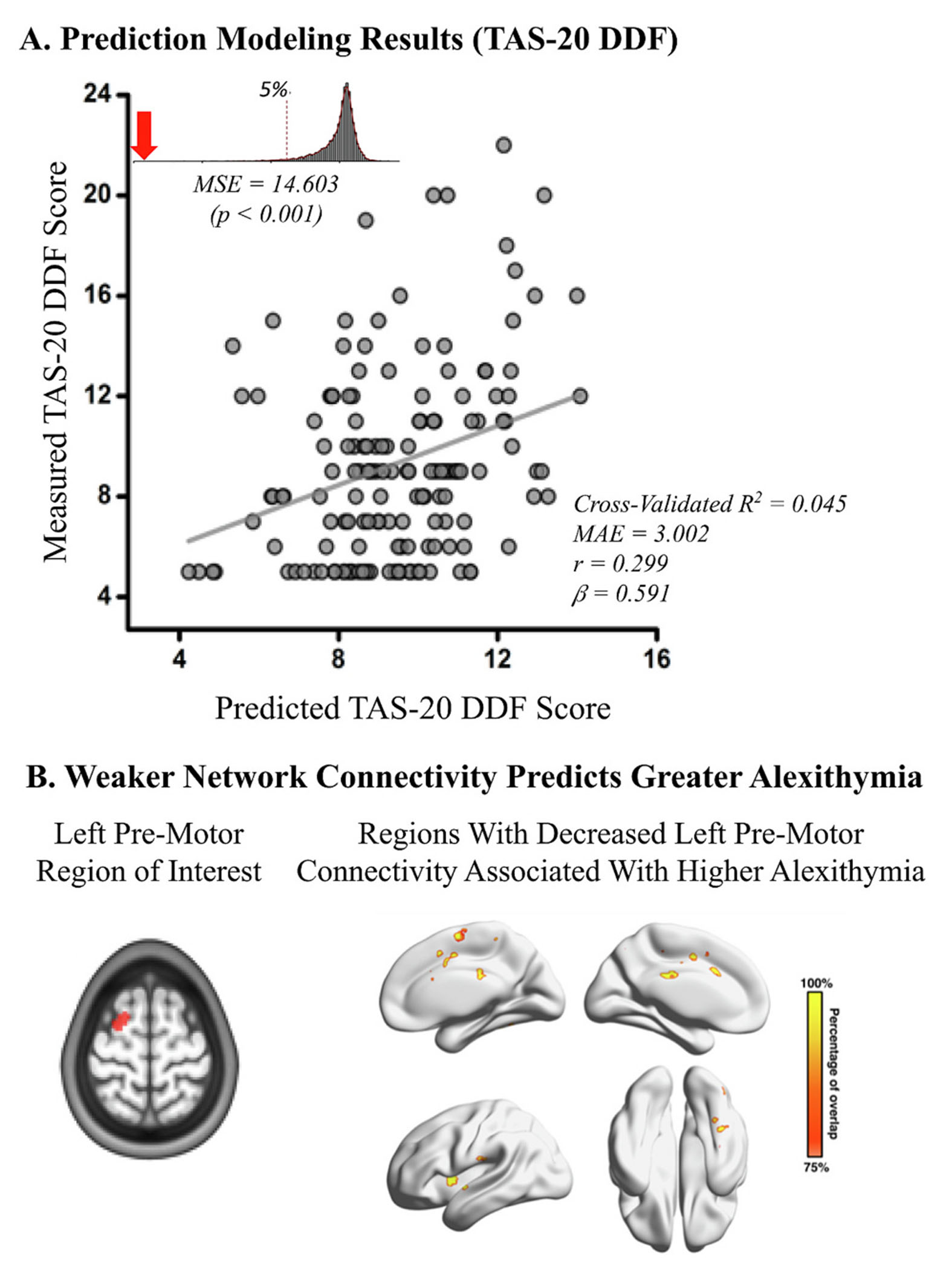
Prediction modeling and functional circuits predicting the TAS-20 subscale DDF. Panel A shows the statistical significance of the final prediction model via permutation, and the prediction performance assessed with a linear regression between the predicted vs. the measured TAS-20 DDF subscale. Panel B shows the surviving ROI as well as its functional circuit that underlies the prediction model.

Similarly, for the DDF subscale, the optimal prediction model minimized the MSE between predicted and measured DDF scores. The optimal model was achieved when the network size threshold ranged from 2550 to 3650 voxels, resulting in the best combination of ROIs. The model significantly predicted DDF score (p < 0.001); linear regression of predicted versus observed scores yielded *r* = 0.299, β = 0.591, cross-validated R^2^ of 0.045 and MAE of 3.002 (Fig. 3A). The final DDF model included a premotor cortex (PMC; BA6) network. Higher DDF scores were predicted by reduced connectivity between the PMC and regions of the salience network, such as the aIns and the dACC (Fig. 3B).

## 4. Discussion

Distinct brain networks predictive of alexithymia were identified in a group of healthy participants. Two networks predicted the TAS-20 total score, while a third predicted the DDF subscale. This study is the first to demonstrate that alexithymia is not driven by a single brain region or network, but rather by distributed networks that integrate sensory, affective, and cognitive information. These findings support the view that emotional awareness and processing, core components of alexithymia, rely on multiple systems for perceiving, experiencing, and understanding emotional states.

Regarding the TAS-20 total score, two independent networks were identified: one involving the right thalamus and another involving the left dlPFC (BA46). These networks likely support the integration of visceral information underlying emotion and the cognitive manipulation of such information, respectively. The thalamus, which is extensively interconnected with the rest of the brain, integrates sensorimotor input with higher-order cortical regions to support a broad range of cognitive and affective functions (Ward, 2013). The findings indicate that individuals with higher levels of alexithymia exhibit reduced connectivity between the thalamus and both the pIns and the sensorimotor cortex, regions critical for integrating visceral and sensorimotor signals. The pIns plays a central role in processing interoceptive and visceromotor information (Craig, 2002), receiving somatosensory input via the thalamus (Centanni et al., 2021; Cerliani et al., 2011; Craig, 2010; Namkung et al., 2017) and guiding behavior in response to internal bodily states (Gehrlach et al., 2019). More broadly, the insula is essential for emotional awareness (Craig, 2002, 2009) and exhibits a functional gradient in which the pIns encodes fundamental somatosensory input, which is further integrated with limbic and cognitive information in the middle insula and aIns (Craig, 2009). The importance of pIns–sensorimotor connectivity for emotional processing is further supported by findings of reduced connectivity and emotional dysfunction in autism, a disorder marked by impaired emotional processing (Ebisch et al., 2010). Consistent with theories emphasizing the role of bodily signals in emotional experience (Craig, 2002; Damasio et al., 1994; James, 1994; Lange, 1922), diminished connectivity between the pIns and primary sensorimotor regions may disrupt the integration of interoceptive information with higher-order processing, thereby contributing to emotional disturbances associated with alexithymia.

Higher total TAS-20 scores were also associated with reduced connectivity between the dlPFC and several brain regions involved in sensorimotor processing, social cognition, language, and memory. This pattern suggests that alexithymia may result from impaired integration of information with higher-order brain regions, such as the dlPFC. Reduced connectivity was observed between the dlPFC and cerebellar lobules VI and VII. Although the cerebellum is traditionally associated with motor coordination, these regions have been increasingly implicated in higher-order affective and cognitive processes through their connectivity with prefrontal and limbic circuits (Stoodley and Schmahmann, 2009, 2010). For instance, individuals with major depressive disorder exhibit reduced functional connectivity between the cerebellum and prefrontal cortex, including the dlPFC, and this reduction has been linked to anhedonia and emotional blunting (Alalade et al., 2011; Wang et al., 2023). Similarly, decreased dlPFC connectivity with sensorimotor areas, including the precentral and postcentral gyri and FEFs, may disrupt attentional orienting, bodily awareness, and motor planning. These processes are known to support emotion-related action selection and awareness (Banker and Tadi, 2023; Jung et al., 2022; Pouget, 2014), and these regions have been implicated in emotion-related psychopathology (Paulus and Stewart, 2014; Ye et al., 2012; Yu et al., 2025; Zhang et al., 2022).

Reduced connectivity was also observed between the dlPFC and temporoparietal regions, including the MTG, STG, and right TPJ, which are critical for social-emotional processing, language, and theory of mind (Saur et al., 2008; Wang et al., 2024). Dysconnectivity in this circuit, previously reported in individuals with autism and social anxiety (Shafritz et al., 2008; Taren et al., 2017), may impair the ability to interpret social-emotional cues and integrate them into personal emotional experience, an impairment consistent with alexithymia. Additionally, diminished dlPFC connectivity with the hippocampus and parahippocampal gyrus, regions involved in contextual memory and autobiographical recall (Li et al., 2016; Sweatt, 2004), may hinder the anchoring of emotions in past experiences and the formation of coherent emotional narratives, as observed in affective disorders such as depression (He et al., 2019).

Collectively, these findings suggest that alexithymia may reflect disrupted integration within a brain network responsible for bodily awareness, social-emotional interpretation, and memory-based contextualization. Impaired dlPFC connectivity with these functionally distinct yet interrelated regions may underlie the difficulties in perceiving, regulating, and understanding emotions that characterize the alexithymia phenotype.

Beyond the networks associated with the TAS-20 Total score, the DDF subscale was linked to reduced functional connectivity between the left PMC (BA6) and the aIns and dACC. These regions likely collaborate to support the integration of emotional, interoceptive, and cognitive information, which is essential for describing feelings. The PMC, traditionally associated with planning and preparing motor actions (Gallese and Goldman, 1998), also contributes to understanding others’ actions through a mirroring mechanism that links perception and motor representation, thereby facilitating the interpretation of others’ behaviors (Acharya and Shukla, 2012; Bastiaansen et al., 2009). This brain region, together with the aIns and dACC, may ultimately support understanding and describing emotional states in oneself and others. In conjunction with the aIns and dACC, which are key nodes of the salience network, the PMC contributes to integrating salient internal and external information, guiding adaptive responses, and facilitating the translation of emotional states into communicative behavior (Uddin, 2014).

Disrupted connectivity between the salience network and PMC may impair detection and response to emotionally relevant cues, both internal (such as bodily sensations) and external (such as facial expressions or tone of voice), thereby hindering access to and articulation of emotional states (Peters et al., 2016; Schimmelpfennig et al., 2023). In the context of alexithymia, particularly the DDF component, weakened connectivity between the PMC and salience network regions may indicate disruption of neural processes that translate affective experience into expression. These findings suggest that individuals with higher DDF scores may struggle to access and express emotional experiences due to reduced integration between regions linking internal affective states with action planning and communication.

Neuroimaging serves as a powerful research tool for identifying clinically meaningful subtypes within mental health and substance use disorders. These subtypes, defined by differences in brain connectivity or emotional processing, may ultimately inform more personalized and effective treatment strategies, including pharmacological interventions. In this study, a cross-validated prediction model demonstrated that patterns of functional connectivity were associated with individual differences in alexithymia, providing initial support for their potential relevance as candidate biomarkers. If further validated, these connectivity patterns could be used to identify individuals at greater risk for difficulties in emotional awareness and regulation. This approach may help identify individuals more likely to engage in substance use or other avoidance and emotional suppression techniques as maladaptive strategies for coping with vague or undifferentiated negative emotional states. By improving early identification and targeting of these risk profiles, neuroimaging research, particularly predictive modeling, may ultimately contribute to more precise and preventive approaches in clinical care.

Several limitations should be considered when interpreting these findings. First, although the sample size of 149 participants is greater than many fMRI studies, it remains relatively modest for robustly generalizing predictive models. Larger samples are necessary to validate the reproducibility of the connectivity patterns and predictive associations identified. Nevertheless, cross-validated predictive modeling provided additional support for the functional connectivity findings. Predicted TAS-20 Total and DDF subscale scores were significantly correlated with measured scores (*r* = 0.283 and *r* = 0.299, respectively). While these correlation coefficients are modest, they are comparable to rsFC-based predictive modeling studies in affective neuroscience (Wang et al., 2021; Morfini et al., 2025) and the corresponding mean squared errors (MSE = 69.59 for Total; MSE = 14.60 for DDF) were significantly lower than those obtained through permutation testing (*p* < 0.001), indicating that the predictive performance was unlikely to have occurred by chance. These results suggest that the model captured meaningful variance in alexithymia scores based on whole-brain resting-state connectivity and future studies with independent data could provide further validation. Additionally, the cross-sectional design of the study limits inference about temporal directionality and causality; the model captures associations at a single time point and may not generalize to longitudinal or prospective prediction. Future studies should examine alexithymia using longitudinal designs to characterize its temporal dynamics and evaluate predictive validity over time.

Second, this study focused a priori on the TAS-20 total score, as well as the TAS-20 DDF subscale, given it is a reliable and stable measure that is meaningfully linked to psychopathology and underlying brain connectivity (Preece et al., 2017; Sutherland et al., 2022; Liang et al., 2015; de Timary et al., 2008). The DIF and EOT subscales were not included as analytic targets. This targeted approach enables a more precise examination of theoretically relevant aspects of alexithymia, but it restricts conclusions regarding its broader, multidimensional structure. In addition, although the TAS-20 is widely used and well-validated, it remains a self-report measure and is therefore susceptible to biases related to introspective ability and self-awareness, factors particularly relevant in alexithymia. Future research using larger and more diverse samples will be necessary to determine whether DIF and EOT demonstrate distinct or complementary neural correlates and to address limitations inherent in self-report measures. Additionally, although the sample excluded individuals with psychiatric diagnoses, subclinical variation in depressive and anxiety symptoms may still influence these findings. Future studies incorporating dimensional measures of affective symptoms are needed to further disentangle these effects.

Third, in the current prediction study, sex was included as a covariate in all models to account for potential confounding effects; however, the study was not specifically designed to test sex as a moderator. Nevertheless, given that alexithymia and its neural correlates may differ between males and females, we conducted exploratory analyses. These analyses revealed no significant interaction between sex and alexithymia in the voxel-wise degree centrality analysis, nor a significant interaction between sex and predicted alexithymia scores in the prediction model. In addition, no significant differences in alexithymia scores were observed between males and females. Together, these results do not provide evidence for robust sex-related differences in the current sample. However, these analyses were exploratory in nature and should be interpreted with caution. Future studies with more targeted designs and diverse samples are needed to more comprehensively evaluate sex as a potential moderator.

Fourth, several methodological considerations related to feature selection and data acquisition may influence the interpretation of the data. Our cross-validation framework did not include the ROI definition step, which may introduce optimistic bias in predictive performance estimates. While this approach enabled stable and interpretable circuit-level characterization, future studies employing independent datasets for feature selection are needed to validate the robustness and generalizability of these findings. We also used threshold (*p* < 0.005) for feature pre-selection in the predictive modeling. This choice represents a pragmatic balance between identifying interpretable brain circuits and maintaining valid predictive performance. While this threshold is consistent with prior connectome-based predictive modeling approaches (Shen et al., 2017) that pre-select statistically significant brain–behavior associations, future studies with larger sample sizes could further optimize this parameter through additional fine-tuning within nested validation frameworks. In addition, the resting-state fMRI scan of 6 minutes is relatively short by contemporary standards for functional connectivity analyses. Although prior work has demonstrated that meaningful connectivity patterns can be derived from scan durations of this length, reliability is known to improve with longer acquisitions (Birn et al., 2013; Van Dijk et al., 2010). Future studies using longer scan durations may help to further validate and strengthen the robustness of the current findings.

Finally, it may be unexpected that the amygdala was not indicated in any of the predictive models. Since the amygdala plays a central role in processing and regulating emotional stimuli, such as facial expressions, and in assigning emotional significance to experiences to guide appropriate behavioral responses (Adolphs and Spezio, 2006), its involvement might be anticipated in the networks reported. However, several methodological and sample-related differences may explain this finding. Prior studies identifying amygdala involvement often used a priori region-of-interest analyses or tasks that explicitly engaged emotional processing. In contrast, the present analyses were conducted using resting-state fMRI, which does not involve direct engagement with emotionally salient cues, likely reducing the likelihood of observing amygdala responsivity. Additionally, the sample consisted of healthy adults without a psychiatric diagnoses and subclinical levels of alexithymia, which may have further limited the detection of amygdala-related neural changes. It is possible that significant amygdala activation or connectivity would be more readily observed in a similar exploratory study in populations with clinically significant levels of alexithymia. Although amygdala activation was not observed, prior research has shown that the amygdala connects with the dlPFC (Connolly et al., 2017), thalamus (Hu et al., 2014), and PMC (Gilboa et al., 2004) in the context of emotion regulation. Thus, the amygdala may still play a modulatory role in the broader neural networks implicated in these findings, even if not directly observable in the present data. Importantly, these results move beyond a focus on a single brain region, advancing the field by emphasizing that alexithymia is predicted by distributed dysconnectivity between cortical and subcortical regions.

## 5. Conclusion

Overall, higher levels of alexithymia in healthy individuals are associated with reduced connectivity between brain networks involved in interoception, emotion regulation, and executive function. This reduced connectivity likely contributes to core features of alexithymia, such as diminished awareness and articulation of emotional states. By clarifying the neural basis of alexithymia, this study provides a foundation for disentangling the contributions of alexithymia and other co-occurring mental health disorders to emotional dysregulation. Understanding the neural underpinnings of alexithymia, independent of comorbidities, is essential for refining treatment strategies that address each condition and their interactions more effectively.

## Supplementary Material

MMC1

## Figures and Tables

**Table 1:** Demographic, imaging, and the alexithymia assessments.

	HA group (*n* = 149)
Age (years)	31.37 ± 10.03 (18–58)
Sex (M/F)	68 / 81
Race	
African American	82
American Indian/Alaska Native	1
Asian	13
White	48
Mixed	5
TAS-20 Total	36.58 ± 8.57
TAS-20 DIF	9.27 ± 3.47
TAS-20 DDF	9.30 ± 3.92
TAS-20 EOT	18.01 ± 4.18
Mean head motion (mm)	0.07 ± 0.03

Abbreviations: HA, healthy adult; TAS, Toronto Alexithymia Scale; DIF, difficulty identifying feelings; DDF, difficulty describing feelings; EOT, externally oriented thinking.

**Table 2:** Seed ROIs identified from the correlation between the whole brain degree centrality and TAS-20 Total score.

Brain region	MNI coordinates (LPI)			Cluster size (mm^3^)
	X	Y	Z	
L-Insula	−34.5	1.5	15.5	8192
L-Putamen	−24	7	−7	
R-Thalamus^a^	9.5	−18.5	7.5	3648
L-Thalamus	−11	−14	7	
L-MCC	−6.5	−10.5	43.5	2368
L-Cerebellum(VIII)	−22.5	−66.5	−52.5	1920
R-MCC	9.5	5.5	39.5	1408
Precuneus	1.5	−58.5	59.5	1408
L-SPL	−22.5	−62.5	55.5	1088
L-Cerebellum(VI)	−14.5	−74.5	−20.5	1024
L-dlPFC^a^	−34.5	37.5	31.5	960
R-Cerebellum(VIII)	13.5	−62.5	−56.5	896
R-Cerebellum(VIII)	33.5	−50.5	−52.5	832
R-IFG	57.5	13.5	15.5	832
R-dlPFC	41.5	45.5	15.5	832

Abbreviations: MCC, middle cingulate cortex; SPL, superior parietal lobule; dlPFC, dorsolateral prefrontal cortex; IFG, inferior frontal gyrus.

a ROIs with significant prediction.

**Table 3:** Seed ROIs identified from the correlation pattern between whole brain degree centrality and TAS-20 DDF subscale.

Brain region	MNI coordinates (LPI)			Cluster size (mm^3^)
	X	Y	Z	
L-Insula	−34.5	1.5	15.5	2240
MCC	9.5	5.5	39.5	2176
L-PMC^a^	−22.5	1.5	67.5	1792
R-IFG	61.5	5.5	7.5	1728
L-LinG	−18.5	−58.5	−4.5	1472
L-dlPFC	−38.5	37.5	31.5	1280
L-IFG	−62.5	−2.5	11.5	1152
R-SFG	33.5	−6.5	63.5	960
SMA	5.5	−2.5	67.5	896

Abbreviations: MCC, middle cingulate cortex; PMC, premotor cortex; dlPFC, dorsolateral prefrontal cortex; SFG, superior frontal gyrus; IFG, inferior frontal gyrus; LinG, lingual gyrus; SMA, supplementary motor area.

a ROIs with significant prediction.

## Data Availability

Data or materials for the experiments are available upon request, and the experiment was not preregistered.

## Appendix A. Supplementary data

Supplementary data to this article can be found online at https://doi.org/10.1016/j.jad.2026.122208.
