# Supplementary material for: Resting-state functional connectivity and alexithymia: Preliminary predictive evidence
Source: J Affect Disord. Author manuscript; Available in PMC 2026 Aug 6. (PMC13446112; doi:10.1016/j.jad.2026.122208)
Supplement: Supplementary file 1 [file NIHMS2197205-supplement-MMC1.pdf]

Supplementary material for

Resting-state functional connectivity and alexithymia: preliminary predictive evidence

Anika Holton, B.S.; Tianye Zhai, Ph.D.; Elise Shealy, B.S.; Samuel Lucero, B.S.; Laura Murray, Ph.D.; Kevin Noemer, M.A.; Yihong Yang, Ph.D.; Betty Jo Salmeron, M.D.; Amy Janes, Ph.D.

Content list:

Figure S1 Schematic flow chart of the analytical pipeline.

Figure S2 Whole brain degree-centrality correlation pattern with TAS-20.

Table S1 Distribution of number of usable TRs after censoring.

Table S2 Relationship between prediction error and the number of usable TRs after censoring.

Table S3 Mean absolute error (MAE) and the cross-validated R^2 of prediction models.

Table S4 Sex – Alexithymia interaction in voxel-wise degree centrality interaction, sex – predicted Alexithymia interaction, and sex differences in Alexithymia scores.

Figure S1 Schematic flow chart of the analytical pipeline.

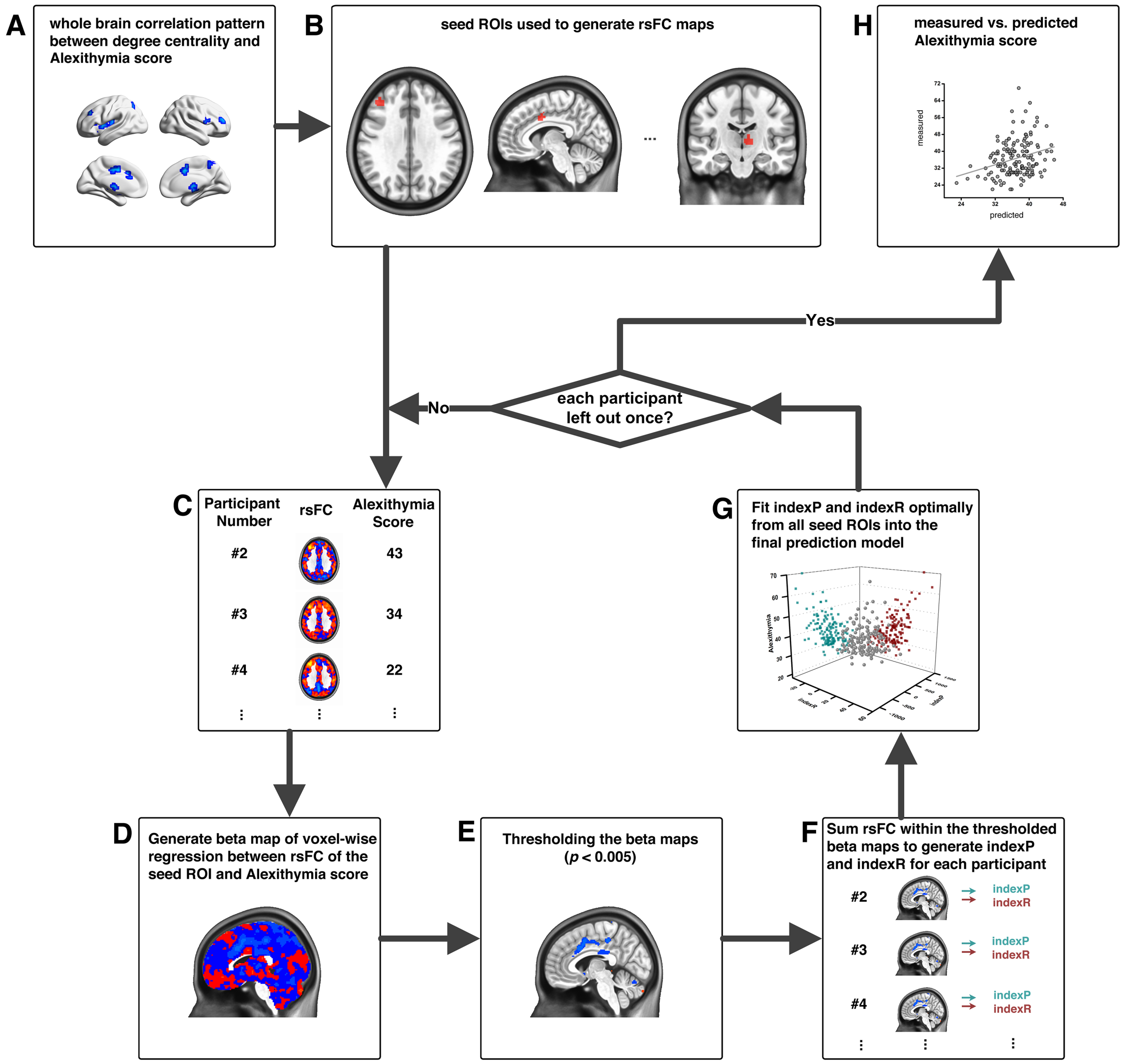

Multiple brain regions are identified with whole brain correlation pattern between the Alexithymia scores and the degree centrality **(A)**; each of these regions are serving as seed ROI for subsequent functional connectivity analysis **(B)**; the whole-brain rsFC of each seed is calculated for each participant **(C)**; a voxel-wise regression is conducted between the rsFC of a specific seed ROI and the Alexithymia scores generating beta maps **(D)**; thresholding the beta maps (p<0.005) **(E)**; generation of composite indices indexP and indexR by linearly summation of the rsFC within the thresholded beta maps for the negative and positive beta voxels, respectively **(F)**; construction of the final prediction model by fitting indexP and indexR optimally from all seed ROIs **(G)**; the steps from C to G were conducted in a cross-validation framework (leave-one-out), the final prediction model was evaluated by comparing the predicted Alexithymia scores to the measured ones **(H)**.
Abbreviations: ROI, region-of-interest; rsFC, resting-state functional connectivity.

Figure S2 Whole brain degree-centrality correlation pattern with TAS-20.

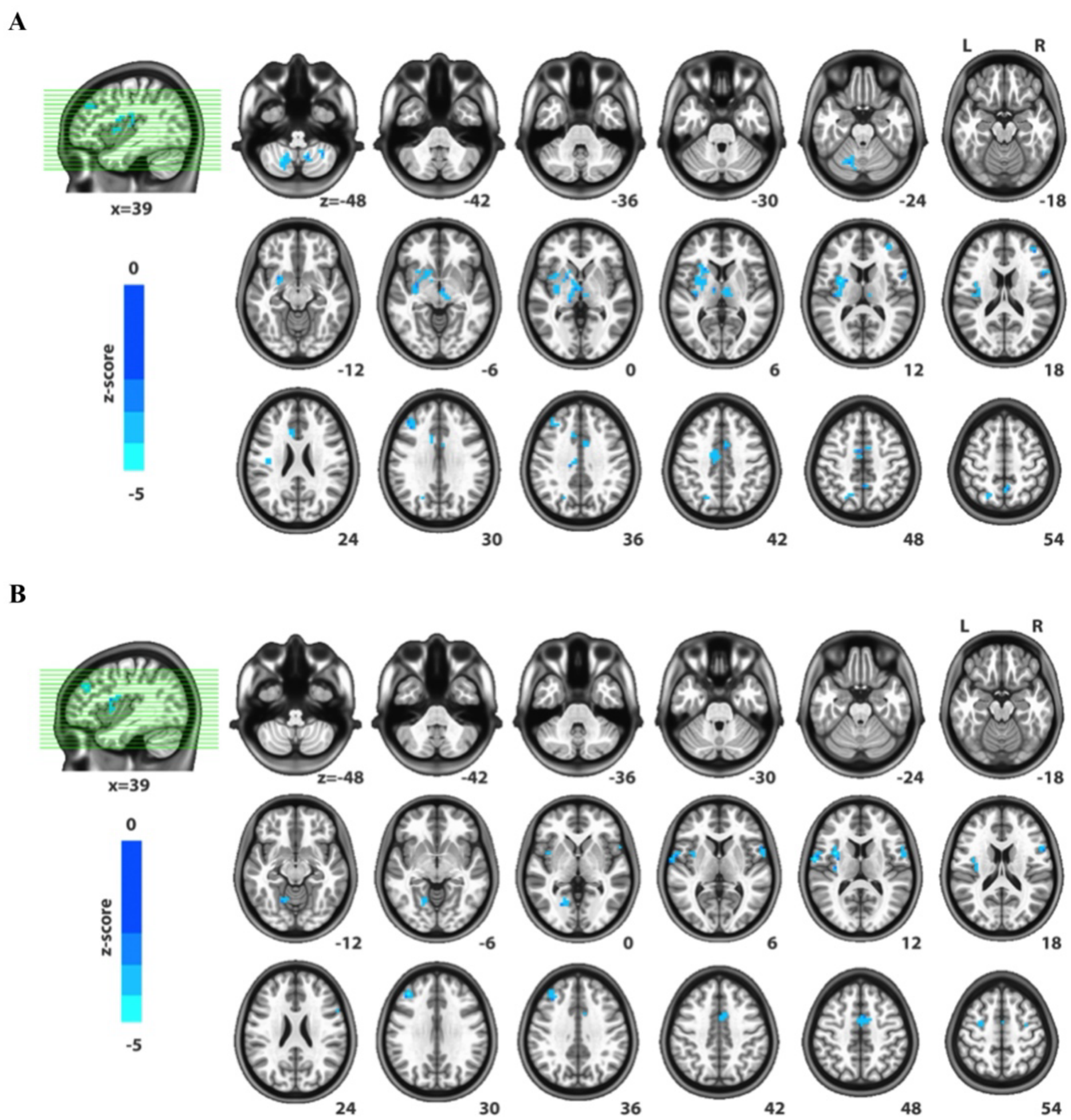

The whole brain degree-centrality correlation pattern with TAS-20 Total score **(A)**; and the whole brain degree-centrality correlation pattern with TAS-20 DDF subscale **(B)**. Voxel-wise correlation was thresholded with voxel level $p<0.001$, cluster level $\alpha<0.05$, corrected. *Abbreviations:* TAS, Toronto Alexithymia Scale; DDF, difficulty describing feelings.

Table S1 Distribution of number of usable TRs after censoring.

	Mean	Sd	Range
No. of usable TRs after censoring	174.013	6.568	139-177

Table S2 Relationship between prediction error and the number of usable TRs after censoring.

Model	Pearson Correlation	Spearman Correlation
TAS-20 total	r = -0.071, p = 0.387	r= -0.005, p = 0.953
TAS-20 DDF	r = -0.053, p = 0.520	r= -0.025, p = 0.764

Table S3 Mean absolute error (MAE) and the cross-validated R^2 of prediction models.

Model	MAE	Cross-validated R^2
TAS-20 total	6.768	0.047
TAS-20 DDF	3.002	0.045

Table S4 Sex – Alexithymia interaction in voxel-wise degree centrality interaction, sex – predicted Alexithymia interaction, and sex differences in Alexithymia scores.

Model	Voxel-wise degree centrality (sex – Alexithymia interaction)	Sex – predicted Alexithymia interaction	Sex difference in Alexithymia scores
TAS-20 total	n.s.	β = 0.007, p = 0.985	$t(143.69) = -0.044$, $p = 0.965$
TAS-20 DDF	n.s.	β = 0.067, p = 0.833	$t(141.43) = -0.186$, $p = 0.853$
